# The Impact of High‐Intensity Exercise in Normobaric Hypoxia on Right Ventricular Function in Trained and Untrained Men—An Echocardiographic Study

**DOI:** 10.1002/cph4.70090

**Published:** 2026-01-06

**Authors:** Gajda Robert, Czuba Miłosz, Płoszczyca Kamila, Kowalik Ewa, Niemaszyk Adam, Starczewski Michał, Grzebisz‐Zatońska Natalia, Kaczmarczyk Katarzyna, Langfort Józef

**Affiliations:** ^1^ Department of Kinesiology and Health Prevention Jan Dlugosz University Czestochowa Poland; ^2^ Center for Sports Cardiology at the Gajda‐Med Medical Center in Pultusk Pultusk Poland; ^3^ Faculty of Rehabilitation Józef Piłsudski University of Physical Education in Warsaw Warsaw Poland; ^4^ Department of Congenital Heart Diseases National Institute of Cardiology Warsaw Poland; ^5^ Department of Sports Theory The Jerzy Kukuczka Academy of Physical Education Katowice Poland

## Abstract

Acute exposure to hypoxia affects the cardiovascular system, especially pulmonary circulation and right heart hemodynamics. However, the impact of normobaric hypoxia on the right heart chambers during exercise is still not clear. This study examined whether a single bout of high‐intensity exercise to voluntary exhaustion under acute moderate normobaric hypoxia (~3000 m a.s.l.; FiO_2_ = 14.4%) induces significant changes in right ventricular (RV) and right atrial (RA) dimensions or RV systolic function compared to normoxia in trained and untrained men. Twenty‐four healthy males (12 trained cyclists, 12 untrained) completed randomized trials involving exhaustive exercise under normoxic and hypoxic conditions. Echocardiographic assessments were conducted at rest and post‐exercise. While hypoxia was found to reduce total mechanical work, end‐exercise heart rate and oxygen saturation in both groups, no differences were observed in the post‐exercise RV response between normoxia and hypoxia. Only untrained men showed increased resting RV dimensions and fractional area change (FAC) in hypoxia. Both groups exhibited post‐exercise declines in tricuspid annular plane systolic excursion (TAPSE), systolic tissue Doppler velocity (*S*′ wave), and right atrial area (RAA), but no additive effect of hypoxia was observed. These results indicate that acute moderate normobaric hypoxia does not impose additional RV load during maximal exercise in healthy athletes and untrained men.

**Trial Registration:** ClinicalTrials.gov: NCT06896773

## Graphical abstract

A single bout of high‐intensity exercise to volitional exhaustion under acute normobaric hypoxia (~3000 m a.s.l.; FiO_2_ = 14.4%) does not induce greater alterations in right ventricular systolic function compared to normoxia in either trained or untrained healthy men.

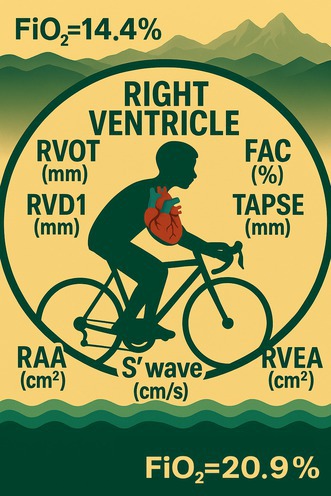

## 1 Introduction

In sports science and practice, training in both hypobaric hypoxia (decreased atmospheric pressure) and normobaric hypoxia (oxygen dilution with nitrogen) has become standard protocol across various disciplines. These methods aim to enhance exercise capacity at sea level, or to facilitate acclimatization prior to competition at or ascending to high altitude (Płoszczyca et al. 2018). In recent years, there has also been growing attention in the therapeutic applications of normobaric hypoxia, particularly in the prevention and management of chronic non‐communicable diseases and in improving quality of life among patients and older adults (Millet et al. 2016). Exposure to hypoxic conditions, both at rest and during physical exertion, activates numerous adaptive mechanisms in the body, seeking to restore homeostasis. These physiological responses can enhance the efficacy of both traditional training and therapeutic methods (Niemaszyk et al. 2025).

Acute exposure to hypoxia is a significant stressor for the cardiovascular system, and its most pronounced effects are observed mainly in the pulmonary circulation. Acute hypoxic exposure leads to a resting increase in pulmonary artery resistance and subsequently to an increase in pulmonary vascular resistance (PVR) and pulmonary artery pressure (PAP). Pulmonary blood pressure rises immediately after exposure to hypoxia and peaks at about 5 min after exposure (Talbot et al. 2005). For instance, breathing a gas mixture containing 11% oxygen has been shown to increase PAP by 56% (16 vs. 25 mmHg; Zhao et al. 2001).

It is known that physical exercise under normoxic conditions already imposes additional stress on the pulmonary circulation, as the elevated stroke volume is not matched by sufficient pulmonary vascular distension, leading to an increase in PAP (Naeije and Chesler 2012). When exercise is performed under hypoxic conditions, this effect can be further exacerbated, resulting in even higher PAP and increased wall stress of the right ventricle (RV). These conditions raise the risk of exercise‐induced cardiac fatigue (Naeije and Chesler 2012; Naeije and Dedobbeleer 2013).

Under hypoxic conditions, the combination of increased cardiac output and elevated RV load significantly increases the oxygen demand of the heart muscle. This, in turn, predisposes the RV to dysfunctional impairment. Several interrelated factors contribute to this risk, including hypovolemia, increased blood viscosity, activation of the sympathetic nervous system, and heat stress (Naeije 2010). These factors require functional and geometric adaptation of the RV to maintain heart function and oxygen delivery to tissues.

Most available data, obtained primarily from population and epidemiology studies, indicate a protective effect of prolonged hypobaric hypoxia on the myocardium. However, some studies have also reported adverse effects under such conditions (Ostadal and Kolar 2007). Additionally, previous research has shown that exposure to hypoxia, or physical exercise performed under such conditions, may reduce the risk of cardiovascular diseases and support cardiac rehabilitation (Niemaszyk et al. 2025). However, it is important to distinguish that cardiac rehabilitation differs substantially from sports training in terms of exercise load, for example, intensity, volume, and the training methods used. What is more, little is so far known about the impact of high‐intensity exercises conducted in hypoxic conditions on the physiological state of the myocardium in trained and untrained healthy men. In the clinical setting of athletes, tests to volitional exhaustion are commonly used to test the cardiovascular response to maximum physical exercise.

Previous investigations have shown an increase in myocardial damage biomarkers after both acute prolonged endurance exercise and high‐intensity exercise in normoxia (König et al. 2003; Wedin and Henriksson 2015; Weippert et al. 2016; Li et al. 2020). Weippert et al. (2016) reported elevated cardiac biomarkers following high‐intensity exercise in normoxia, while echocardiographic indices of myocardial function in healthy active men indicated normal cardiac function. In a recent study, Goliniewski et al. (2024) found no changes in the activity of cardiac markers—including troponin I and T, heart‐type fatty acid‐binding protein (H‐FABP), creatine kinase‐MB isoenzyme (CK‐MB), and myoglobin (Mb)—following a single bout of intense interval exercise and a 4‐week period of high‐intensity training under normobaric hypoxia (F_i_O_2_ = 15.5%).

There is little existing literature addressing the effects of a single bout of high‐intensity exercise performed during acute exposure to hypoxia on the dimensions and function of the right ventricle (RV) and right atrium (RA). Consequently, the myocardial response to hypoxia remains poorly studied, and the available data are inconclusive. Importantly, research in this area has clear practical relevance: high‐intensity training units carried out in moderate hypoxia (F_i_O_2_ = 16.5%–14.4%, ~2000–3000 m a.s.l.) are currently standard in many sports disciplines (Wilber 2007; Cheung 2010; Millet et al. 2010; Czuba et al. 2011, 2017, 2019; Faiss et al. 2013), and consequently, in the case of possible detection of adverse changes in the heart could have clinical importance.

Thus, the purpose of this study was to determine changes in the RV and RA dimensions and RV function in response to a single session of exercise performed to volitional exhaustion under both normoxic and normobaric hypoxic conditions in both trained and untrained men. Based on our previous research, we hypothesized that (1) high‐intensity exercise performed to voluntary exhaustion under acute normobaric hypoxia (F_i_O_2_ = 14.4%, ~3000 m a.s.l.) would not cause greater changes in RV and RA dimensions and RV function compared to normoxia, in either untrained or trained men, and (2) due to different adaptations of the cardiovascular system, the RV response to hypoxia and exercise would be different in untrained men and endurance trained athletes.

## 2 Materials and Methods

### 2.1 Study Participants

The study included 24 healthy male participants divided into two groups: 12 untrained males (UT group; aged 31.7 ± 8.3 years; body height 181.1 ± 6.5 cm; body mass 85.7 ± 11.5 kg; fat content (%) 17.3% ± 6.3%; VO_2max_ 44.1 ± 7.4 mL·kg^−1^·min^−1^) as well as 12 trained male athlete–cyclists (T group; aged 26.5 ± 7.7 years; body height 180.8 ± 3.6 cm; body mass 71.4 ± 5.3 kg; fat content (%) 12.3% ± 2.6%; VO_2max_ 64.2 ± 2.9 mL·kg^−1^·min^−1^). The inclusion criteria for both groups were as follows: (1) age 20–40 years; (2) no chronic diseases; (3) systolic blood pressure 100–140 mmHg and diastolic blood pressure 60–90 mmHg. The exclusion criteria, in turn, were (1) use of drugs, alcohol consumption, or smoking; (2) hypertension; (3) premature termination of the exercise test.

**FIGURE 1:**
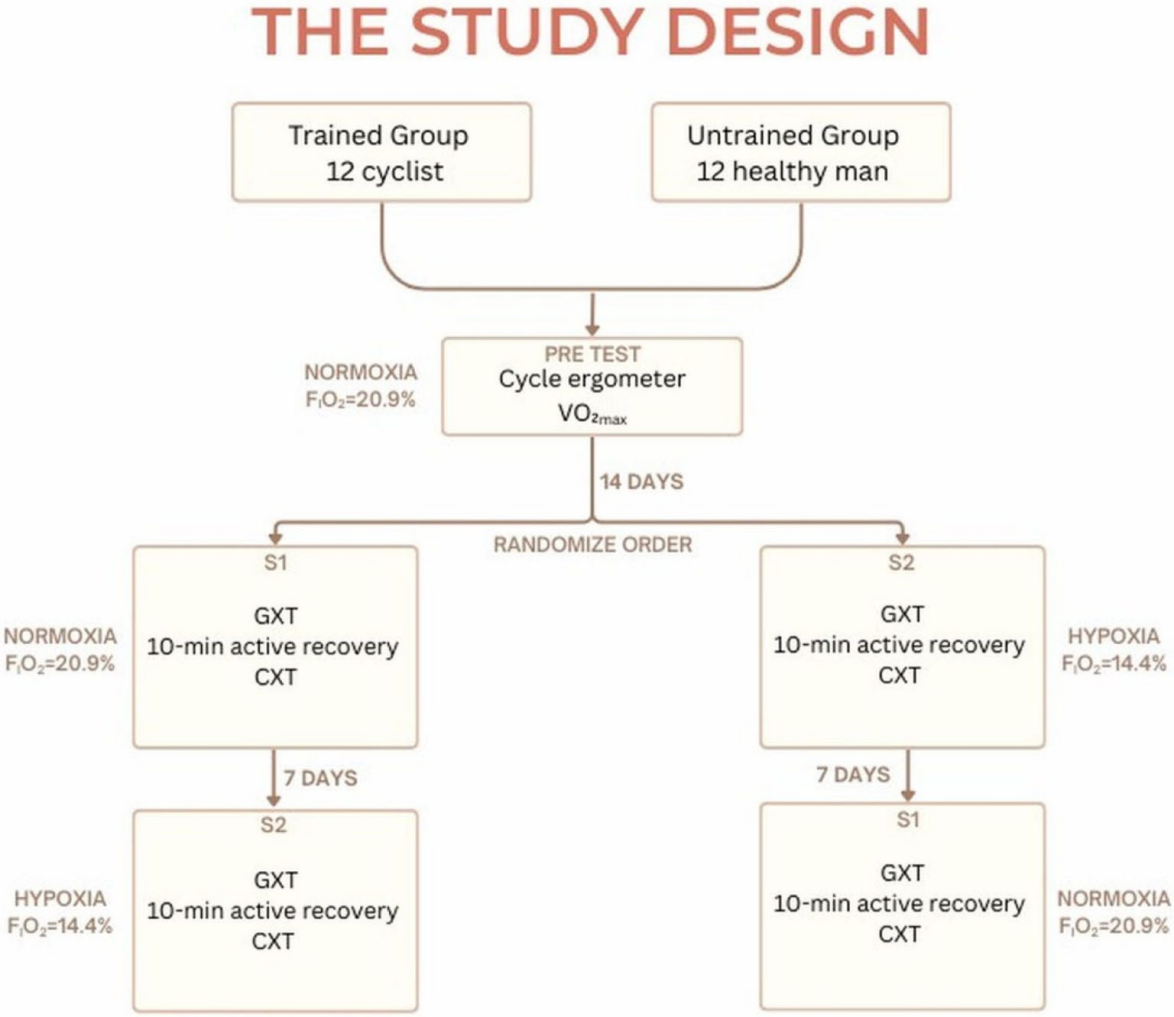
Illustration of the study design. CXT, constant‐workload exercise test at the individual lactate threshold workload, continuing until volitional exhaustion; GXT, graded exercise test to volitional exhaustion; VO_2max_, maximal oxygen uptake.

Prior to the experiment, a preliminary test was conducted in which all participants performed an incremental test on a cycle ergometer under normoxic conditions (Figure 1). This test was used to determine VO_2max_ and to familiarize the participants with the test protocol. All subjects provided current medical certificates confirming their good health and ability to perform intensive physical exercise. Additionally, all the subjects were informed of the purpose of the study and provided written consent to participate. The study adhered to the principles outlined in the Declaration of Helsinki and was approved by the Bioethics Committee at the University of Zielona Góra, Poland (Resolution No. 21/2022 of November 9, 2022).

### 2.2 Study Design

The experiment consisted of two test series that differed in terms of the conditions: S1 (normoxia) and S2 (normobaric hypoxia) (Figure 1). The fraction of inspired oxygen (FiO_2_) differed for the two series: for S1 (normoxia), FiO_2_ = 20.9%, while S2 (3000 m a.s.l.), FiO_2_ = 14.4%. The order of the series was randomized for each participant. All tests were performed in a normobaric hypoxic chamber (AirZone, AirSport, Poland), but participants were not informed about the conditions actually present in the chamber (nomoxia or hypoxia). Each series was carried out over a single day and included two consecutive ergocycle tests, separated by a 10‐min active recovery period (see Section 2.3; Figure 1). The methodology was consistent across both series, and all testing sessions were performed at the same time of day to minimize circadian variability. A 1‐week active recovery period separated the two series; during this time, participants avoided hypoxic exposure and refrained from high‐intensity exercise.

### 2.3 Testing Protocol

In series S1, body mass and body composition were taken between 7:00 and 7:30 a.m., before breakfast. Body height was measured using an anthropometer with an accuracy of 0.5 cm, and body composition was estimated using bioelectrical impedance analysis (InBody 220, Biospace).

In both test series (S1, S2), 2 h after consuming a light mixed meal (5 kcal/1 kg body weight; 50% carbohydrates, 30% fat, 20% protein) and after 20 min of passive exposure to the selected conditions (normoxia or hypoxia), participants performed two consecutive ergocycle tests, separated by a 10‐min active recovery period. All tests were performed on a cycle ergometer (Excalibur Sport, Lode BV) adjusted individually to each participant.

In each series, the first test was a graded exercise test (GXT) to volitional exhaustion. The exercise began with a workload of 90 W, increasing by increments of 30 W every 3 min until the participant could no longer continue. At the end of each load (the last 15 s) capillary blood samples were obtained from the fingertips in order to determine blood lactate levels (LA; LABTREND, BST Bio Sensor Technology GmbH, Germany). These data were used to analyze the kinetics of LA concentration in the blood and to evaluate the individual lactate threshold using the Dmax method (Cheng et al. 1992).

After the first test, a 10‐min active recovery period was performed, maintaining 30% of the maximum workload reached during the test until volitional exhaustion (WR_max_). Next, participants performed a constant‐workload exercise test (CXT) at their individual lactate threshold workload (WR_LT_), continuing until volitional exhaustion. During the CXT, the participants were allowed to drink water ad libitum. Before and immediately after completion of the CXT, capillary blood was obtained from the fingertip to determine the LA concentration and acid–base balance. Additionally, HR and SpO_2_ were continuously monitored at rest, as well as throughout the GXT and CXT (WristOx2 pulse oximeter, Nonin Medical Inc.).

### 2.4 Transthoracic Echocardiography

In both series (S1, S2), participants underwent two complete transthoracic echocardiographic (TTE) examinations: after 20 min of passive exposure to the selected assigned condition (normoxia or hypoxia) and the second immediately after the two consecutive ergocycle tests. All echocardiographic assessments were performed using the EPIQ system (Philips Medical Systems, Andover, MA, USA) and an X5‐1 phased‐array transducer, with the participant in the left lateral decubitus position. Cardiac measurements were obtained by an expert echocardiographer (K.E.) following the American Society of Echocardiography guidelines for the assessment of the right heart (Rudski et al. 2010).

**FIGURE 2:**
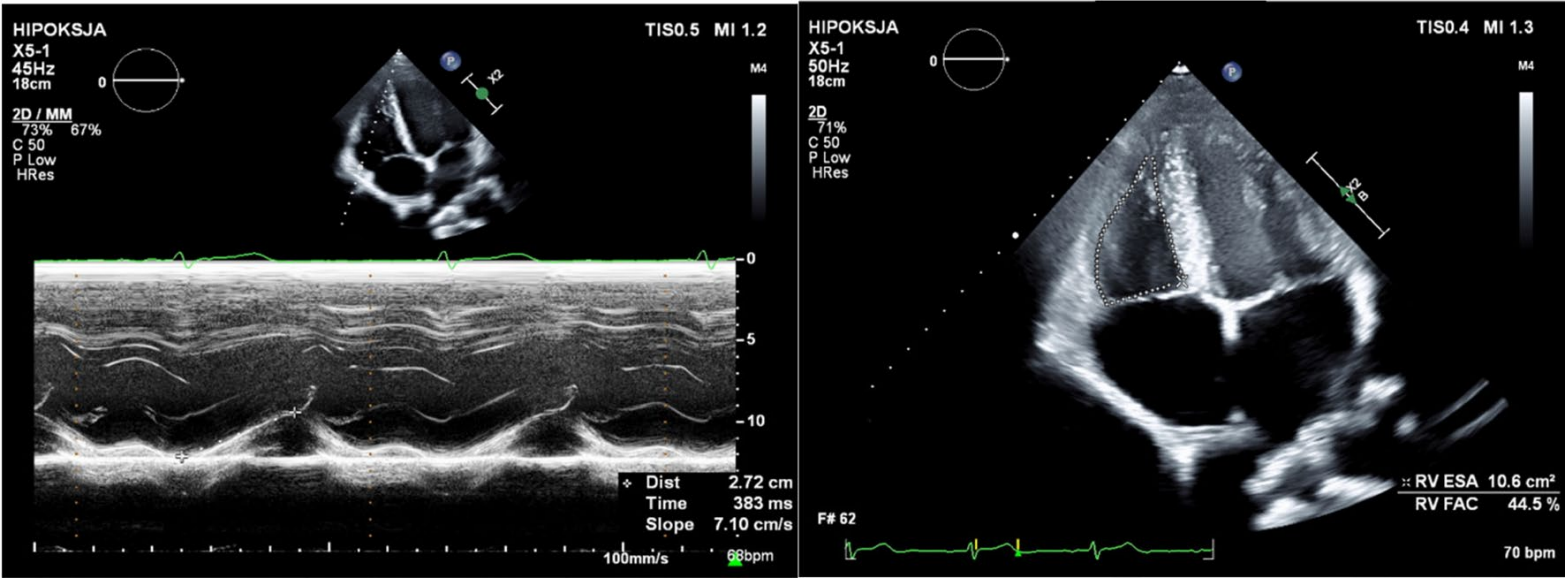
Examples of echocardiographic assessment of the RV function in an untrained participant at rest. Left panel—measurement of tricuspid annular plane systolic excursion (TAPSE) using the M‐mode method (TAPSE: 27 mm). Right panel—tracing of the RV endocardial border in the apical 4‐chamber view in end‐systole, with a calculated fractional area change (FAC) of 44.5%.

RV dimensions were assessed at end‐diastole, using the parasternal view for the right ventricular outflow tract (RVOT) and RV‐focused apical four‐chamber view for the RV basal dimension (RVD1). From the latter view, RV end‐diastolic and end‐systolic areas were traced and RV fractional area change (FAC) was calculated (Figure 2). The tricuspid annular plane systolic excursion (TAPSE) was acquired by the conventional M‐mode method at the lateral tricuspid annulus (Figure 2). Pulsed tissue Doppler with a sample volume placed at the lateral corner of the tricuspid annulus was used to assess its velocity (*S*′ wave). The right atrial area (RAA) was measured from the apical four‐chamber view at the end of ventricular systole.

### 2.5 Statistical Analysis

Data were collected and processed using Statistica software (StatSoft). Before performing statistical analysis, the Shapiro–Wilk test was used to test the normal distribution of variables and Levene's test was used to check the homogeneity of variance. If the assumptions for parametric testing were met, a two‐way repeated‐measures ANOVA was performed. To determine the significance of differences between specific combinations of means (UT vs. T under the same conditions; rest vs. post‐exercise under the same conditions within each group; normoxia vs. hypoxia within each group), contrast analysis was employed. Statistical significance was set at *p* < 0.05 for all comparisons.

## 3 Results

### 3.1 Changes in Mechanical Work, Heart Rate and Saturation of Hemoglobin During Exercise to Exhaustion Under Normoxia and Hypoxia

The mechanical work during both tests (GXT + CXT, *W*_mech_) significantly reduced (*p* < 0.01) in both groups under hypoxic conditions—by 31.5% in the UT group and 18% in the T group. *W*_mech_ was significantly higher (*p* < 0.001) in the T group compared to the UT group, again in both normoxia (36% higher) and hypoxia (43%). In both groups, hypoxia induced a significant decrease (*p* < 0.001) in heart rate recorded at the end of exercise performed to exhaustion (HR_end_), by approx. 4.5%. In addition, values of the HR_end_ in the T group were significantly higher (*p* < 0.05) than in the UT group in both conditions, but when these values were converted to a percentage of maximum heart rate for the given conditions (%HR_max_con), no significant differences were observed between groups and conditions. Under hypoxia, there was also a significant reduction (*p* < 0.001) in hemoglobin saturation registered at the end of exercise (SpO_2end_) in both the UT (12%) and T (10%) groups. No significant changes in delta values of LA (∆LA) or pH (∆pH) in response to exercise to exhaustion were observed between conditions or groups (Table 1).

**TABLE 1:** Mechanical work, heart rate and saturation of hemoglobin during exercise to exhaustion under normoxia and hypoxia in the untrained (UT) and trained (T) group. All values shown as mean ± SD.

Variable	Group	Normoxia	Hypoxia (3000 m)
*W*_mech_ (kJ)	UT	534.62 ± 173.96	434.94 ± 144.37***
	T	937.83 ± 183.14^###^	762.06 ± 165.57**^,###^
*W*_mech_ (kJ/kg)	UT	6.28 ± 1.92	5.12 ± 1.6***
	T	13.08 ± 2.06^###^	10.71 ± 2.41**^,###^
HR_end_ (bpm)	UT	172.41 ± 8.71	164.41 ± 7.58***
	T	179.33 ± 7.41^#^	171.75 ± 8.51***^,#^
%HR_max_con	UT	95.65 ± 2.72	96.07 ± 2.96
	T	95.35 ± 2.23	95.48 ± 2.57
SpO_2end_ (%)	UT	96.16 ± 1.11	84.91 ± 2.39***
	T	94.16 ± 1.11	84.41 ± 4.37***
∆LA (mmol/L)	UT	6.51 ± 1.9	6.35 ± 2.35
	T	5.17 ± 1.72	4.75 ± 1.72
∆pH	UT	−0.114 ± 0.048	−0.101 ± 0.041
	T	−0.091 ± 0.026	−0.074 ± 0.042

Abbreviations: %HR_max_con, % of maximum heart rate under the given conditions; ∆LA, change in blood lactate concentration in response to exercise; ∆pH, change in pH in response to exercise; HR_end_, heart rate recorded at the end of exercise performed to exhaustion; SpO_2end_, hemoglobin saturation registered at the end of exercise; *W*_mech_, total mechanical work performed during both tests (GXT + CXT).

***p < 0.001, **p < 0.001—significant differences between normoxia and hypoxia. ^###^ *p* < 0.001, ^#^ *p* < 0.05—significant differences between groups (UT vs. T) under given conditions.

### 3.2 Echocardiographic Parameters of RV and RA Dimensions and RV Function

In the UT group, the right ventricular outflow tract (RVOT) diameter at rest was 6.4% larger (*p* < 0.05) in hypoxia compared to normoxia. Additionally, the resting values of RVOT diameter in hypoxia in the UT group were also significantly larger (*p* < 0.05) compared to the T group. Similar changes were observed in resting values of fractional area change (FAC) in the UT group, which also increased significantly, by 9.7%, under hypoxia compared to normoxia. Under normoxia conditions, the resting values of FAC were significantly lower (*p* < 0.05), by 24.8%, in the UT group compared to the T group. Under hypoxia, there were no significant differences in resting values of this variable.

In both groups (UT and T), exercise to volitional exhaustion caused significant changes in values of FAC, tricuspid annular plane systolic excursion (TAPSE), systolic velocity (RV *S*′ wave), and right atrial area (RAA), as shown by ANOVA results (Table 2). Under normoxia, there was a significant decrease (*p* < 0.05) in FAC immediately after exercise, by 14.2% (*p* < 0.05), in the T group only. Additionally, the delta values of FAC (∆FAC) were significantly lower (*p* < 0.01) in the T group compared to the UT group only in normoxia. No statistically significant changes in FAC or ∆FAC were observed for the T group in hypoxia or for the UT group in either condition.

**TABLE 2:** Echocardiographic parameters of RV and RA dimensions and RV function at rest and post‐exercise until volitional exhaustion under normoxia and hypoxia in the untrained (UT) and trained (T) group. All values shown as mean ± SD.

Variable	Group	Normoxia		Hypoxia (3000 m)		ANOVA results	
		At rest (*x* ± SD)	Post ex. (*x* ± SD)	At rest (*x* ± SD)	Post ex. (*x* ± SD)	Exercise	Exercise and conditions
RVOT (mm)	UT	32.46^$^ ± 2.72	33.96 ± 2.89	34.54 ± 3.35^$^	33.92 ± 3.31	*F* = 0.627 *p* = 0.436	*F* = 3.701 *p* = 0.067
	T	31.50 ± 4.61	29.96 ± 4.11	31.01 ± 3.49^#^	31.12 ± 3.81	*F* = 0.937 *p* = 0.343	*F* = 1.243 *p* = 0.276
RVD1 (mm)	UT	40.83 ± 3.04	40.33 ± 5.63	40.08 ± 5.38	38.25 ± 6.03	*F* = 1.653 *p* = 0.211	*F* = 0.540 *p* = 0.047
	T	40.51 ± 2.93	35.42 ± 11.35	41.45 ± 4.34	39.98 ± 3.80	*F* = 3.638 *p* = 0.069	*F* = 1.243 *p* = 0.276
RVEDA (cm^2^)	UT	24.48 ± 4.78	22.94 ± 6.36	24.28 ± 7.33	22.71 ± 7.65	*F* = 3.548 *p* = 0.072	*F* = 1.109 *p* = 0.303
	T	25.67 ± 4.43	23.63 ± 3.16	25.06 ± 4.56	22.65 ± 3.24	*F* = 6.309 *p* = 0.019	*F* = 0.045 *p* = 0.834
FAC (%)	UT	42.76 ± 7.29	47.23 ± 10.70	46.93 ± 5.34^$^	49.84 ± 8.22	*F* = 4.679 *p* = 0.041	*F* = 0.211 *p* = 0.650
	T	53.38 ± 6.03^###^	45.77 ± 6.95*	51.78 ± 8.49	48.12 ± 7.07	*F* = 8.242 *p* = 0.008	*F* = 1.013 *p* = 0.325
TAPSE (mm)	UT	27.16 ± 3.52	22.50 ± 4.77*	27.17 ± 4.17	22.00 ± 5.24*	*F* = 18.819 *p* = 0.001	*F* = 0.050 *p* = 0.824
	T	26.31 ± 2.75	19.85 ± 3.37***	26.57 ± 4.68	20.07 ± 3.37***	*F* = 83.228 *p* = 0.001	*F* = 0.001 *p* = 0.981
*S*′ wave (cm/s)	UT	14.21 ± 1.89	12.27 ± 1.21*	15.29 ± 2.22	12.42 ± 2.03**	*F* = 23.574 *p* = 0.001	*F* = 0.895 *p* = 0.354
	T	14.22 ± 1.23	10.96 ± 1.50***	14.75 ± 1.97	12.06 ± 2.32**	*F* = 44.745 *p* = 0.001	*F* = 0.411 *p* = 0.528
RAA (cm^2^)	UT	20.95 ± 3.68	19.40 ± 3.98*	21.33 ± 4.14	18.88 ± 3.15*	*F* = 13.628 *p* = 0.001	*F* = 0.674 *p* = 0.420
	T	21.63 ± 2.70	19.98 ± 3.52*	22.73 ± 3.58	20.80 ± 2.61*	*F* = 10.498 *p* = 0.003	*F* = 0.066 *p* = 0.800

Abbreviations: FAC, values of fractional area change; RAA, right atrial area; RVD1, right ventricular basal diameter; RVEDA, right ventricular end‐diastolic area; RVOT, right ventricular outflow tract; *S*′ wave, systolic velocity; TAPSE, tricuspid annular plane systolic excursion.

***p < 0.001, **p < 0.001, *p < 0.05—significant differences between at rest and post exercise under given conditions. ^###^ *p* < 0.001, ^#^ *p* < 0.05—significant differences between groups (UT vs. T) under given conditions. ^$^ *p* < 0.05—significant differences between values at rest under normoxia and hypoxia.

**TABLE 3:** Comparison of the delta (∆) values of selected variables in response to exercise to exhaustion, between untrained and trained men (NT vs. T group), under normoxic and hypoxic conditions—the table presents only those ∆ values for which there were significant differences.

Variable	Group	Normoxia	Hypoxia (3000 m)
∆RVOT (mm)	UT	1.5 ± 1.60	−0.62 ± 3.47
	T	−1.53 ± 3.20^##^	0.10 ± 3.97
∆FAC (%)	UT	4.47 ± 9.82	2.90 ± 6.58
	T	−7.60 ± 6.12^##^	−3.65 ± 12.13
∆*S*′ wave (cm/s)	UT	−1.68 ± 2.03	−2.86 ± 2.95
	T	−3.68 ± 1.80^#^	2.69 ± 2.49

Abbreviations: FAC, values of fractional area change; RVOT, right ventricular outflow tract; *S*′ wave, systolic velocity.

^#^ *p* < 0.05, ^##^ *p* < 0.01—T vs. UT.

**FIGURE 3:**
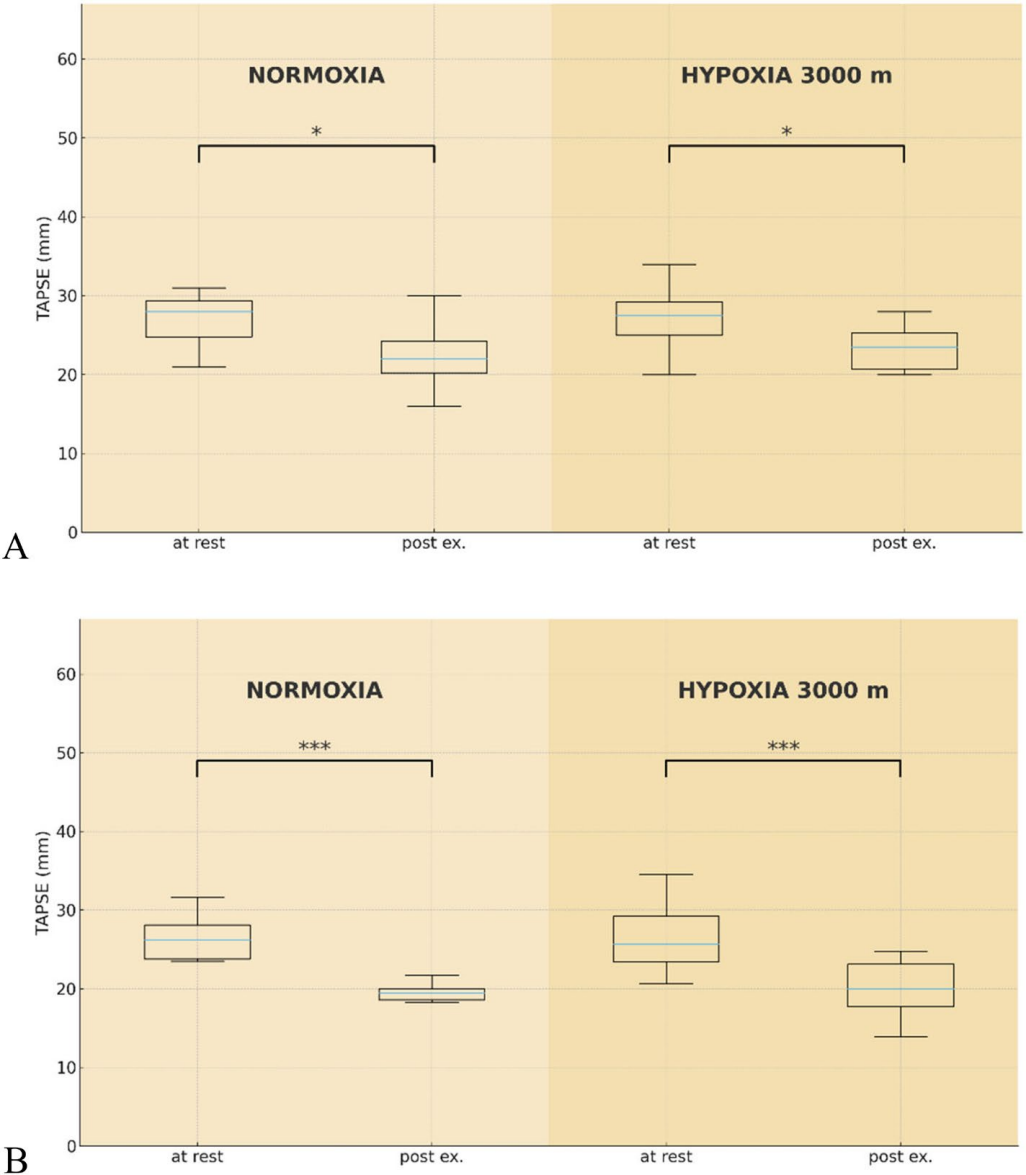
Tricuspid annular plane systolic excursion (TAPSE) at rest and post‐exercise in untrained (A) and trained (B) participants under normoxic and hypoxic (3000 m) conditions; ***p < 0.001, *p < 0.05—significant differences between at rest and post exercise under given conditions.

In the UT group, TAPSE decreased significantly (*p* < 0.05) post‐exercise, by 17.1% in normoxia and by 19% in hypoxia. Similar changes occurred in the T group, where TAPSE after exercise decreased (*p* < 0.001) by 24.5% in normoxia and by 24.4% in hypoxia (Figure 3). The *S*′ wave also decreased significantly (*p* < 0.05) after exercise in both groups. Under normoxia, the *S*′ wave declined significantly (*p* < 0.05), by 13.6%, in the UT group and by 22.9% (*p* < 0.001) in the T group. After exercise in hypoxia, the *S*′ wave decreased by approx. 18% in both groups (*p* < 0.01). However, the delta values for the *S*′ wave (∆*S*′) were significantly lower (*p* < 0.01) in the T group compared to the UT group, but only under normoxic conditions (Table 3).

In both groups, exercise until volitional exhaustion significantly reduced RAA values (*p* < 0.05), by approx. 7.5% in normoxia. Under hypoxia, RAA decreased post‐exercise (*p* < 0.05) by 11.5% in the UT group and by 8.5% in the T group (Table 2). Notably, although RVOT values did not change significantly in response to exercise in either group, the T group exhibited significantly lower delta RVOT values after exercise in normoxia (Table 3).

## 4 Discussion

Our study was directed whether intense, prolonged exercise to volitional exhaustion under normobaric hypoxia affects right ventricular (RV) and right atrial (RA) structure and RV and function differently in untrained and trained men than when compared to the same exercise under normoxia. No evidence was found of significantly greater RV workload in response to intense exercise to exhaustion under hypoxia compared to normoxia, in either group, thus there is no evidence of an acute negative effect of moderate hypoxic conditions (FiO_2_ = 14.4%) on RV myocardium in healthy untrained and trained males.

Acute hypoxia induces pulmonary vasoconstriction and increases pulmonary artery pressure, which can have implications for RV structure and function (Mamazhakypov et al. 2022). Our results showed that even after just 20 min of passive exposure to moderate normobaric hypoxia, a measurable enlargement of the RV outflow tract (RVOT) was observed in the untrained group (+2.1 mm). This finding comports with previous research by Netzer et al. (2017), who demonstrated a progressive increase in RVOT diameter under hypoxic conditions (11% O_2_, equivalent to ~4500 m altitude), with changes of +4 mm after 30 min and +13 mm after 150 min of resting exposure.

We also observed an augmented RV systolic function in response to hypoxia in the untrained group, as reflected by an increase in fractional area change (FAC) of +4.2%. Previous studies involving healthy subjects exposed to hypoxia have reported similar trends in RV FAC, although these changes did not always reach statistical significance (Kjaergaard et al. 2007; Seccombe et al. 2017). Conversely, other studies have found no significant changes in RV FAC (Pavelescu and Naeije 2012; Pezzuto et al. 2018; Ewalts et al. 2021). These discrepancies may be attributable to variations in study design, duration of exposure, and timing of echocardiographic assessments during high‐altitude or hypoxic conditions. Moreover, some studies included both women and men, which limits the possibility of direct comparison with our findings. According to recent literature (Lasocka‐Koriat et al. 2024), the mechanisms of cardiac adaptation to exercise differ between males and females. The authors highlight sex‐specific differences in left ventricular remodeling, autonomic regulation, biomarker profiles, and electrocardiographic patterns.

Acute normobaric hypoxia induced an increase in RVOT diameter and FAC only in untrained individuals. No changes were observed in resting RV and RA dimensions or RV function in trained athletes in response to hypoxic exposure. This differing response between trained and untrained participants likely reflects differences in cardiovascular system adaptation. It is probable that the hypoxia with an FiO_2_ level of 14.4% applied in this study did not constitute a sufficiently strong stimulus to induce changes in the dimensions of right heart chambers or RV systolic function in trained individuals.

Although acute hypoxic exposure increases RV hemodynamic load, it is important to note that just 1 min of reoxygenation can reduce the impact of hypoxia on RV systolic pressure by nearly half. Moreover, in healthy individuals, the vascular system is capable of rapidly compensating for hypoxic pulmonary vasoconstriction, effectively reversing RV dilation (Netzer et al. 2017; Ewalts et al. 2021).

Our study found that endurance athletes exhibited significantly higher resting RV FAC values under normoxic conditions compared to untrained individuals (53.4% vs. 42.8%). Similar findings were reported by Pagourelias et al. (2013) in a study involving a large cohort of top‐level Caucasian male athletes. In their study, the resting FAC averaged 50.4% in endurance athletes, while in the untrained control group it was 42.7%. La Gerche et al. (2011) also reported RV FAC values exceeding 50% in endurance‐trained athletes. It is important to note that despite these elevated values, FAC remained within the normal reference range (Rudski et al. 2010). Higher FAC values reflect an overall enhancement in right ventricular contractility among trained individuals. Systematic training induces physiological, structural, and functional adaptations of the myocardium (D'Andrea et al. 2003; Kasikcioglu et al. 2005). Due to the hemodynamic overload associated with long‐term endurance training, increases are typically observed in the size of both left and right heart chambers, left ventricular wall thickness, and indices of both systolic and diastolic function—both at rest and during exercise—when compared to non‐athletes (D'Andrea et al. 2014; Parry‐Williams and Sharma 2020; Flanagan et al. 2023).

Not only long‐term training, but also a single bout of exercise has been found to induce changes in myocardial structure and function. During intense physical activity, the relative increase in pressure within the pulmonary circulation exceeds that of the systemic circulation (Kovacs et al. 2009; Argiento et al. 2010; La Gerche et al. 2010), and when combined with elevated cardiac output, this leads to greater right ventricular (RV) stress (La Gerche et al. 2011). Argiento et al. (2010) demonstrated that exercise to volitional exhaustion is accompanied by a fourfold increase in cardiac output and an elevation in systolic pulmonary artery pressure (sPAP) exceeding 40 mmHg. The RV response to exercise depends on the type, intensity, and duration of the physical effort (La Gerche et al. 2012; Coates et al. 2020). Endurance exercise has been shown to cause an acute reduction in RV systolic function, as evidenced by changes in echocardiographic indices such as a decrease in right ventricular ejection fraction (RVEF), RV fractional area change (FAC), and tricuspid annular plane systolic excursion (TAPSE) (Ramcharan et al. 2024). In our study, we observed that intense exercise to exhaustion resulted in decreased longitudinal systolic function of the RV, indicated by reductions in TAPSE and the RV *S*′ wave, as well as a decrease in right atrial area (RAA). These changes occurred regardless of training status and environmental condition (normoxia vs. hypoxia). Several previous studies have also reported significant post‐exercise reductions in TAPSE, RV *S*′ wave, and RAA following endurance exercise under both normoxic and hypoxic conditions (Elliott and La Gerche 2015; de la Garza et al. 2016; Coates et al. 2020; Kleinnibbelink et al. 2021). We also found that trained athletes experienced a post‐exercise reduction in RV FAC under normoxic conditions, a change not observed in untrained individuals. This finding aligns with earlier studies showing a significant association between training status and RV FAC, with athletes training more hours per week exhibiting a greater acute reduction in RV FAC following endurance exercise (Ramcharan et al. 2024).

It is widely accepted that acute RV dysfunction is caused by RV afterload during endurance exercise, resulting from a sharp rise in pulmonary artery systolic pressure. Increased RV afterload leads to greater RV wall stress, which in turn contributes to an acute reduction in ventricular contractility (Ramcharan et al. 2024). It has been observed that the longer the duration of exercise, the more pronounced the post‐exercise impairment of RV function (La Gerche et al. 2012; de la Garza et al. 2016). Based on this, it has been suggested that the heart has a finite capacity to sustain elevated workloads during prolonged physical activity (La Gerche et al. 2012). However, more recent studies by Coates et al. (2020) have challenged the notion that cardiac fatigue is primarily dependent on exercise duration. Instead, they propose that exercise intensity may be a more critical factor influencing changes in myocardial function. Our findings contribute a new dimension to this discussion. We demonstrated that the RV response to exercise under normoxic conditions differed significantly between the UT and T groups, with athletes exhibiting a more pronounced reduction in RV function (Table 3). This difference most likely resulted from the nearly twofold higher amount of mechanical work performed during exercise by the T group compared to the UT group (13.08 vs. 6.28 kJ/kg). These results suggest that the overall workload—defined as a combination of exercise duration and intensity—may be a key determinant of post‐exercise RV dysfunction.

One important finding of our study is the demonstration that moderate normobaric hypoxia (~3000 m a.s.l.) does not alter the RV response to exercise and does not impose additional strain on the right ventricle. Similar conclusions were drawn in a recent study by Forbes et al. (2024), who, based on direct hemodynamic measurements of the right ventricle and pulmonary artery, found that RV function during exercise remains preserved in response to acute hypoxia (FiO_2_ = 12%) despite elevated pulmonary arterial pressures. However, these findings should be interpreted and compared with caution due to the small sample size (*n* = 10) and the inclusion of both women and men, without accounting for potential sex‐related differences in RV function. Notably, in both studies, exercise intensity was appropriately adjusted to the environmental conditions (based on lactate threshold or VO_2_max determined under hypoxia). Earlier, Kleinnibbelink et al. (2021) reported that normobaric hypoxia (FiO_2_ 14.5%, ~3000 m) had no effect on the extent of RV fatigue induced by a 45‐min high‐intensity running session (85% HR_max_) in healthy individuals. Our findings complement this body of evidence, showing that hypoxia is also safe for trained athletes performing substantially higher workloads under hypoxic conditions compared to untrained individuals. In the T group, the mechanical work performed during hypoxic exercise was twice as high as in the UT group. Despite this, exercise in hypoxia did not impose greater strain on the RV in athletes compared to normoxia. We suspect that in trained individuals, intense exercise to volitional exhaustion under hypoxia leads to pronounced local muscular fatigue (Romer et al. 2007; Amann and Dempsey 2016) which ultimately causes exercise cessation and thus protects the heart from excessive exercise‐induced RV dysfunction. Interestingly, in athletes, the reduction of RV systolic function following exercise in hypoxia was smaller than after normoxic exercise—an inverse pattern compared to untrained individuals (Table 3).

## 5 Limitations

Our study has several limitations. First, the relatively small sample size limits the generalizability of the findings. As the study population consisted exclusively of healthy males aged 20–40 years, the results cannot be directly extrapolated to females, other age groups, or individuals with pre‐existing medical conditions. Second, due to the absence of a complete tricuspid regurgitation Doppler jet in most participants, we were unable to reliably estimate RV systolic pressure. Consequently, we did not analyze the hemodynamics of the right ventricle or pulmonary circulation. The impact of different exercise durations on the echocardiographic parameters cannot be ruled out in our study either. Lastly, while regional differences in RV myocardial systolic function may exist, we did not assess RV myocardial deformation (longitudinal strain) due to the high variability associated with this measurement and the requirement for additional specialized software.

## 6 Conclusions

The findings of this study have important practical implications, particularly given the widespread use of hypoxic training across many sports disciplines. We found that a single bout of high‐intensity exercise to volitional exhaustion under acute moderate normobaric hypoxia (~3000 m a.s.l.; FiO_2_ = 14.4%) does not induce greater alterations in right ventricular (RV) or right atrial (RA) dimensions or RV systolic function compared to normoxia in either trained or untrained healthy men. Furthermore, the reduction in RV systolic indices post‐exercise was smaller in hypoxia than in normoxia among trained athletes.

## Author Contributions

Conceptualization: G.R., C.M., and L.J. Methodology: G.R., C.M., P.K., and K.E. Validation: K.E. and N.A. Formal analysis: G.R., C.M., and K.K. Investigation: C.M., K.E., N.A., S.M., G.‐Z.N., and K.K. Data curation: C.M., P.K., K.E., N.A., S.M., and G.‐Z.N. Writing – original draft: G.R., C.M., P.K., K.E., and L.J. Writing – review and editing: C.M., P.K., K.E., N.A., and L.J. Supervision: G.R. and L.J. Project administration: C.M. and K.K. Funding acquisition, C.M. All authors have read and agreed to the published version of the manuscript.

## Funding

This work was supported by the grant no. NdS‐II/SP/0431/2024/01 from the Ministry of Science and Higher Education, Poland.

## Ethics Statement

The study was conducted according to the guidelines of the Declaration of Helsinki and approved by the Bioethics Committee at the University of Zielona Góra, Poland (No. 21/2022 of November 9, 2022).

## Conflicts of Interest

The authors declare no conflicts of interest.

## Supporting information


**Data S1:** cph470090‐sup‐0001‐DataS1.xlsx.

## Data Availability

The data that supports the findings of this study are available in the Supporting Information of this article.
